# Recent advances in expression and purification strategies for plant made vaccines

**DOI:** 10.3389/fpls.2023.1273958

**Published:** 2023-11-23

**Authors:** Srividhya Venkataraman, Imran Khan, Peyman Habibi, Maria Le, Rory Lippert, Kathleen Hefferon

**Affiliations:** ^1^ Department of Cell and Systems Biology, University of Toronto, Toronto, ON, Canada; ^2^ Forte Protein, Center for Life Science Ventures, Cornell University, Ithaca, NY, United States; ^3^ Department of Food Science, Cornell University, Ithaca, NY, United States; ^4^ Department of Microbiology, Cornell University, Ithaca, NY, United States

**Keywords:** plant biotechnology, expression, purification, molecular farming, infectious disease

## Abstract

Plants have been explored as a platform to produce pharmaceutical proteins for over 20 years. Important features such as the cost-effectiveness of production, the ease of scaling up to manufacturing capacity, the lack of cold chain requirements and the ability to produce complex therapeutic proteins which are biologically and functionally identical to their mammalian counterparts, make plants a strong alternative for vaccine production. This review article focuses on both the expression as well as the downstream purification processes for plant made vaccines. Expression strategies including transgenic, transient and cell suspension cultures are outlined, and various plant tissues targeted such as leaves and seeds are described. The principal components used for downstream processing of plant made vaccines are examined. The review concludes with a reflection of the future benefits of plant production platforms for vaccine production.

## Introduction

Infectious diseases are the foremost source of human mortalities around the globe. The recent COVID-19 pandemic vividly illustrates the lack of global preparedness for novel viral diseases (Lobato Gomez et al., 2021). In addition to the emergence of newly emerging diseases, many well-known infectious viruses, for instance, Severe Acute Respiratory Syndrome Coronavirus-2 (SARS-CoV-2) and influenza, continuously undergo genetic changes, thus giving rise to frequent outbreaks associated with severe illnesses and fatalities. The rapid and widespread transmission of SARS-CoV-2 has led to an unprecedented global crisis, resulting in millions of deaths (Hu et al., 2021). Therefore, cost effective and rapid biomanufacturing of recombinant vaccine antigens at large scale for remedy against numerous infectious diseases will be urgently needed. Molecular farming, also known as biopharming, is a cutting-edge technology that harnesses the ability of plant’s cells, organs, or tissues as bio-factories for the production of valuable biopharmaceuticals and other high-value proteins, including vaccine antigens (Faye and Gomord, 2010; Ma et al., 2003). This technology offers a promising alternative to traditional methods of protein production using prokaryotic and eukaryotic hosts, for example, *Saccharomyces cerevisiae*, Chinese hamster ovary (CHO) cells, *Escherichia coli (E. coli)*, human embryonic kidney (HEK) cells, *Pichia pastoris* (Cereghino and Cregg, 2000; Macauley-Patrick et al., 2005), and insect systems such as *Spodoptera frugiperda* (Sf9). These production hosts are widely accepted for large scale manufacturing because of the well-defined regulatory pathway available (Schillberg et al., 2005) higher expression and ease of production; however, each production platform comes with its own advantages and disadvantages. Plants are the bio-factories of diverse and complex proteins and offer several advantages. Plants allow for scalable and cost-effective production due to their ability to be cultivated on masses. Plant cells possess the remarkable ability to process proteins post transcription to make tailored glycan structures (post-translational modification) and correct folding, providing an advantage in producing recombinant proteins with specific glycosylation patterns (Gomord and Faye, 2004). As an illustration, plants offer cost-effective and highly scalable production capabilities when compared to CHO cells (Buyel et al., 2017). Transient expression systems, on the other hand, allow for rapid scalability surpassing traditional fermenter-based platforms (Hiatt et al., 2015). Furthermore, in terms of safety, plants pose a lower risk of harboring human or animal pathogens, making them a favorable option for protein production (Nosaki et al., 2021). Thus, the use of plants as production hosts for recombinant vaccines hold immense promise and continues to be an area of active research and development in the field of biotechnology. The selection of an expression host involves considering a blend of host properties, including food or feed crop, ease of genetic manipulation, the presence of specific endogenous metabolic pathways, expression capacity, as well as intellectual property constraints. In addition, product characteristics such as the intended purpose, structural complexity, and the level of purification needed are also crucial factors in choosing a host for production.

Vaccines play a crucial role in preventing and controlling infectious diseases, and their availability and accessibility are of utmost importance for global public health. However, traditional vaccine manufacturing processes often face challenges related to scalability, cost-effectiveness, and cold chain storage requirements. Plant-based systems offer several advantages for vaccine production. Moreover, plants can be engineered to produce complex and multicomponent vaccines, allowing for the production of combination vaccines or those targeting multiple strains of a pathogen. The use of plant systems for vaccine production also offers logistical advantages. Unlike traditional vaccines, plant-based vaccines are stable at higher temperatures and do not require cold chain storage and distribution, making them particularly suitable for resource-limited regions or areas with inadequate infrastructure.

In recent decades, significant strides have been achieved in the advancement of plant-based platforms for vaccine production. Various plant species, including tobacco, maize, potato, and others have been successfully engineered to produce a wide range of vaccines against viral, bacterial, and parasitic diseases. These plant-produced vaccines have shown promising results in preclinical and clinical studies, demonstrating safety, efficacy, and the ability to induce strong immune responses. In this context, here we provide an overview of the current state of vaccine production in plant systems within the field of molecular farming. It discusses the advancements, challenges, and prospects of using plants as bio-factories for vaccine production, highlighting the potential of this innovative approach to revolutionize the vaccine manufacturing landscape and improve global accessibility to life-saving immunizations. Importantly, the shortcomings encountered during expression of recombinant proteins/vaccines in plants are highlighted and the review concludes with a unique perspective and insight into the use of state-of-the-art genome editing technology in circumventing the drawbacks of plant-based expression systems.

## Strategies for expression of vaccines

### Transgenic plants for edible vaccines

Transgenic plants are being explored as a potential platform for producing edible vaccines. Edible seeds or vegetative tissues can be engineered to produce recombinant vaccines that can be administered via the oral route, thus, minimizing the painful needles, and require no cold-chain requirements, enabling rapid and efficient global-scale deployment during vaccine distribution (Stöger et al., 2005; Hefferon, 2014a). The oral route can revolutionize the current approaches for vaccine delivery. Production of vaccine antigen in plant cells has great potential to eliminate prohibitively costly and complex fermentation, simplify its transportation and distribution, eliminate sterile injections and improve the shelf-life for years at ambient temperature (Kurup and Thomas, 2020; Khan and Daniell, 2021). There are several plant species where recombinant vaccines have been expressed to achieve oral delivery such as potato (Mason et al., 1996; Mason et al., 1998), rice (Oszvald et al., 2007; Qian et al., 2008; Fukuda et al., 2018; Saba-Mayoral et al., 2023), banana, tomato (Lou et al., 2007; Davod et al., 2018; Hoshikawa et al., 2019), lettuce (Kim et al., 2007), tobacco (Hahn et al., 2007), alfalfa (Wigdorovitz et al., 1999), wheat (Shi et al., 2023), spinach and carrot.

Plant made vaccines are bioencapsulated by the cell wall and cellular organelles that enable vaccine proteins to withstand degradation and pass through digestive system so that they can be recognized by the immune surveillance system (Khan and Daniell, 2021). Due to its substantial mucosal surface area, the human intestine provides an optimal site of entry for orally administered plant-derived vaccines. The gut-associated lymphoid tissue (GALT), which constitutes the largest immune system tissue in the human body (over 70%), is a crucial reservoir of regulatory T cells (Tregs) and encompasses an extensive surface area of approximately 300 m2 (Vighi et al., 2008). The unique advantage of plant made vaccine antigens is that they become bioencapsulated into plant cell walls; this enables them to withstand the enzymatic and acidic conditions within the gut lumen. Due to the inability of human digestive enzymes to break down the glycosidic bonds of carbohydrates present in the cell wall, plant-made vaccine antigens can successfully pass through the digestive tract. The cellulosome, present in anaerobic cellulolytic bacteria as an extracellular enzyme complex, contains binding, structural, and catalytic domains that enable direct interaction with the plant cell wall and the cleavage of glycosidic bonds. Once the vaccine reaches the gut mucosal layer, the inhabitant bacteria help to break down of the plant cell wall and therefore facilitate the release of vaccine antigen. *Bacteroides fragilis* aids in the penetration of the mucous layer by degrading mucin glycoproteins (Koropatkin et al., 2012). The crossing into the intestinal epithelium and the presentation of vaccine antigens to the immune system is crucial and is accomplished by different tags (receptor binding proteins) attached to the vaccine such as CTB (Sanchez and Holmgren, 2008).

### Transient expression systems

Plant transient expression systems are being utilized as favored expression systems because of their rapid and high production, simplicity for scale up, low cost, ease of purification and most importantly, already established protocols available for some plants (Nosaki et al., 2021). The ability and reliability of transient systems in producing highly valuable and complex proteins has been successfully demonstrated (Lindsay et al., 2018; Malm et al., 2019; Ward et al., 2021). *Nicotiana benthamiana* is a dominant production host for research and commercial purposes that has the potential to make proteins days post-delivery of the DNA-construct, and therefore is utilized for commercial biomanufacturing of hormones, antibodies, enzymes, therapeutics and vaccines etc. by several companies such as Medicago Inc. (https://www.medicago.com), Forte Protein Inc. (www.forteprotein.com), Kentucky BioProcessing (https://kentuckybioprocessing.com) and Icon Genetics (https://www.icongenetics.com). Achieving the highest possible yield is a primary concern in expressing high value proteins, as downstream processing costs rise considerably when proteins are extracted from more diluted mixtures. Transgenic plant development can be a time-consuming project and typically requires around 2-3 years until a homozygous line for a host plant is obtained. In contrast, transient expression enables the swift production of substantial quantities of recombinant proteins. For instance, Medicago Inc., a company utilizing plant-based transient expression systems, can produce the purified end-product of an influenza vaccine just three weeks after receiving the sequence (D’Aoust et al., 2008). Most recently, Medicago Inc. demonstrated the capability of using transient systems for the production of a vaccine against SARS-CoV-2 and influenza (Venkataraman, 2022). The data demonstrated consistent immunogenicity in human clinical trials (Ward et al., 2021). During the recent COVID-19 pandemic, several research groups produced RBD as a vaccine antigen using different host systems. For instance, the Receptor Binding Domain (RBD) of the SARS-CoV-2 spike protein induces humoral immunity in animal models as well as in non-human primates (Diego-Martin et al., 2020; Rattanapisit et al., 2020; Maharjan et al., 2021; Siriwattananon et al., 2021; Ceballo et al., 2022) using *Nicotiana benthamiana* as the production host. Transient expression systems can usually yield recombinant proteins at concentrations of grams per kilogram of fresh biomass and have the potential to improve further with innovations. Several recombinant proteins i.e., vaccines (Lindsay et al., 2018; Malm et al., 2019; Rattanapisit et al., 2020; Ruocco and Strasser, 2022), growth factors (Soleimanizadeh et al., 2022) therapeutics (Gengenbach et al., 2018; Silberstein et al., 2018; Xiong et al., 2022), hormones (Gils et al., 2005; Xiong et al., 2022), and antibodies (Lai et al., 2012) have been produced using transient expression systems. Besides having several advantages over competitive systems, transient expression systems require further innovations with respect to novel vectors, expression strategies, clarification and in particular, downstream processing to achieve the goal of large scale production at a lower cost to make alternative proteins more affordable and accessible.

### Plant suspension culture systems

*In vitro* cultured plant cells, also known as plant cell suspension culture, have shown great promise as bioproduction platforms for alternative proteins. Suspension cultures may be comprised of transgenic plants, hairy roots, protoplasts or cell cultures, and combine the advantages of whole-plant cultivation systems with the benefits of microbial and mammalian cell cultures, making them highly promising and versatile options for producing therapeutic proteins. Scalability, safety, protein folding, post-translational modification, cost of production, stability and flexibility are the key points of consideration for using plant cell suspension culture. Unlike other eukaryotic systems such as CHO or HEK cells, plant cultures can be grown in simple and inexpensive growth media, are easy to scale up for rapid biomanufacturing; both features reduce the overall cost of production. High value recombinant proteins can be secreted by adding a signal peptide to target proteins to simplify downstream processing (Huang et al., 2015). N-terminal signal peptides derived from different sources can transport recombinant proteins into the endoplasmic reticulum (ER) lumen. The transport occurs in a signal recognition particle (SRP)-dependent manner, facilitated by SRP receptors (Gilmore et al., 1982). Subsequently, while residing in the ER, the signal peptides are cleaved from the precursor proteins, and the proteins are then encapsulated into small vesicles, which bud to the Golgi apparatus and ultimately get released into extracellular compartments (Khan et al., 2012). The targeting of recombinant proteins to ER and Golgi apparatus facilitates the correct folding of recombinant proteins, required for their functionality (de Ruijter et al., 2016). Secretory proteins undergo additional modifications in the endoplasmic reticulum (ER) and Golgi apparatus, including N-glycosylation (Faye et al., 2005; Breitling and Aebi, 2013). For the last few decades, several recombinant proteins have been expressed and validated for their functionality. Most importantly, the first plant made recombinant protein taliglucerase alfa that was approved by the FDA was expressed in carrot suspension culture (Tekoah et al., 2015).

Plant cell suspension culture has been used as a host for vaccine production. Most recently, the production of RBD and spike proteins are reported in tobacco cells (BY-2) and Medicago truncatula A17 cells (Rebelo et al., 2022). Considering the advantages of plant suspension culture, numerous vaccine antigens have been successfully expressed and validated for their functionality through both *in-vivo* and *in-vitro* analyses (Smith et al., 2002; Smith et al., 2003; Lenzi et al., 2008; Muthamilselvan et al., 2016). Technology for using cells from carrot, soybean, tobacco, rice, medicago and potato has been developed and have produced vaccines against a variety of diseases. However, there is considerable potential for future research and development (R&D) to optimize the culture environment, with the aim of improving both production levels and protein recovery efficiency. The primary benefits regarding bioreactors are regulated growth environments with complete containment, consistency in protein quality, yield and homogeneity as well as rapid pace of production from the level of gene to protein within a short span 4-5 weeks (Fischer et al., 2009; Huang et al., 2009). Nevertheless, bioreactors are not favorable due to scale-up constraints similar to their mammalian cell culture equivalents such as relatively lower concentrations (Weathers et al., 2010; Xu et al., 2011), instability of secreted protein products (Hellwig et al., 2004), and higher capital expenses compared to greenhouses or open-field cultivation. From the perspective of downstream processing, major advantages of secreted protein products include more facile, low-cost purification (Hellwig et al., 2004; Doran, 2006) along with the drawback that the efficiency of secretion may be circumscribed by protein hydrophobicity, size and/or charge (Fischer et al., 2009). For non-secreted recombinant proteins, plant cell homogenates encounter the same complexities as that of leafy plants. As a result, they may not be as economically attractive as other protein expression systems, as potential savings from producing intracellular proteins are offset when compared to secreted proteins.

### Expression of vaccines and related recombinant proteins from plants

Biotechnological expression of therapeutic proteins requires efficacious expression and purification schemes so that recombinant proteins can be generated in their native conformations. Stable or transient expression of such recombinant therapeutic or vaccine proteins in plants are propitious tools to achieve unbridled potential for both scale-up and reduced production costs (Yusibov and Rabindran, 2008; Phan et al., 2013a). The first proof-of-concept was the expression of Hepatitis B surface antigen (HBsAg) in plants (Mason et al., 1992), following this, several subunit vaccines and virus-like particles have been successfully generated (D’Aoust et al., 2008; Rybicki, 2014; Mbewana et al., 2015). This approach has been recently adopted by Medicago for the generation of the COVID-19 vaccine and seasonal influenza vaccines which have now fortuitously completed clinical trials.

Transient expression systems using plants to biosynthesize recombinant pharmaceutical proteins are found to be advantageous compared to other systems. Transient expression enables rapid scale up compared to any other fermenter-derived platforms (Hiatt et al., 2015; Holtz et al., 2015). Further, transient expression vectors such as those derived from full or deconstructed plant viruses facilitate high level expression of recombinant biopharmaceuticals (Hefferon, 2014b). Nevertheless, two principal challenges must be surmounted to develop plants as expression vehicles for recombinant therapeutics: inadequate expression levels and non-scalable/inefficient purification methods.

### Seed-based expression systems

Seeds have been used for the successful expression of several recombinant proteins i.e., vaccine antigens, therapeutic, antibodies, hormones, industrial enzymes, and cell culture proteins. The USDA has endorsed the field release of transgenic plant seeds expressing proteins such as lactoferrin, serum albumin and human lysozyme in rice, the apolipoprotein in safflower as well as brazzein and hepatitis B surface antigen in corn (http://www.isb.vt.edu/searchrelease-data.aspx). Seed crops present several advantages compared to mammalian cell cultures, transgenic animals and microbial fermentations as host platforms by virtue of the availability of a vast knowledge base regarding their cultivation, harvesting, processing and storage (Kusnadi et al., 1997; Nikolov and Hammes, 2002).

Shi et al., 2023 report the stable expression of the TM-1 gene-encoded amino acid sequences of Mycoplasma gallisepticum (MG), considered as a vaccine antigen candidate against Chronic Respiratory Disease (CRD) affecting chickens. In this study, wheat seed tissues are used as production hosts. The recombinant 41.8 kDa protein was ubiquitously expressed in endosperm tissues and an expression level of 1.03 mg/g dry weight was achieved. Upon oral administration in chickens, this plant-made edible vaccine was effectual in eliciting antibody responses without any identifiable weight loss. Two doses of the orally delivered TM-1 vaccine candidate triggered an immune response and protection against challenge with MG at levels comparable to the commercially produced inactivated vaccine against CRD. This investigation proves that plant-made edible vaccines are safe, scalable, stable at room temperature and cost-effectual.

Shahid et al., 2020 report a study wherein the protective antigens, hemagglutinin-neuraminidase (HN) and fusion (F) proteins of Newcastle Disease Virus (NDV) were expressed using a constitutive seed-specific Zein promoter and the 35S promoter, respectively, in transgenic maize. In this case, almost 2-7.1-fold greater expression of the F gene mRNA was observed in the leaves and about 8-28-fold higher expression of the HN gene mRNA was detected in the seeds. For the F protein, 1.66 µg/ml was observed accounting for 0.5% of the leaf total soluble protein while for the HN protein, 2.4µg/ml was found accounting for 0.8% of the total seed protein. When chicks were orally administered with the seeds and leaves of transgenic maize, an immune response was generated against both NDV antigens.

Stable transgenic rice seeds which express the F protein of Newcastle disease (ND) virus were generated through agroinfiltration (Ma et al., 2020). When this vaccine was inoculated into pathogen-free chickens, it significantly triggered neutralizing antibody responses against both heterologous and homologous ND virus strains. Two doses of 4.5 µg antigen completely protected chickens from challenge with a lethal dose of NDV. Additionally, F protein-immunized chicken exhibited a higher mean weight gain within 15 days following challenge compared to the conventional whole virus vaccine-immunized chickens, thus yielding higher cost benefits. This study underlined the success of plant-based vaccines as NDV eradication platforms.

In the case of nutraceuticals, industrial enzymes and oral vaccines, requirements for processing and purification are minimized through the use of seed crops (Nandi et al., 2002; Yang et al., 2008; Hood and Howard, 2009). In comparison with leaf crops, seed crops contain lesser biomass yields per unit surface area. Nevertheless, if crops are cultivated in open fields, protein stability and economy of scale far outweigh the disadvantage associated with low biomass yield (Nikolov and Hammes, 2002; Schillberg et al., 2005).

### Leaf-based and fruit-based expression platforms

Leafy crops such as tobacco and alfalfa are highly advantageous due to their elevated biomass yields, likelihood of yearly several growth cycles, and well-established infrastructure for agriculture similar to their seed equivalents; however, they have the advantage of lower risk of pollen spread by prevention of flowering. Tobacco is a leading leaf-based platform for commercial expression of recombinant proteins (Dubey et al., 2018; Twyman et al., 2003) ever since the advent of tobacco expressing monoclonal antibody (Hiatt et al., 1989). Additionally, important advances have taken place wherein leaves are used for transient expression of vaccines, monoclonal antibodies and other therapeutics using tobacco (Nicotiana benthamiana) as the expression system (D’Aoust et al., 2010; Joensuu et al., 2010; Conley et al., 2011). Transient expression circumvents regulatory concerns associated with transgenic plants and is currently the preferred method of recombinant protein expression for low-volume protein synthesis (Fischer et al., 2009; Pogue et al., 2010). Transient expression using leafy tissues affords a distinct advantage for tobacco grown in greenhouses particularly in instances where rapid expression of pandemic vaccines is necessary (D’Aoust et al., 2010). The simplicity of leaf-based protein extraction is at times perceived as an advantage compared to extraction from seeds which may necessitate additional operations, for example, soaking and grinding (Schillberg et al., 2005).

Lettuce and tomato are preferred vegetable systems that can be consumed raw (Concha et al., 2017; Miranda et al., 2020). Lettuce (Lactuca sativa) varieties have been used to produce edible vaccines against swine fever, stage I hepatitis and E. coli (Kim et al., 2007). Similarly, carrot (Daucus carota) has been used to obtain edible vaccines against Helicobacter pylori and E. coli (Zhang et al., 2010) Banana (Musa acuminata) has been the plant of choice for raising vaccine against hepatitis B. Banana is consumed raw and is widely cultivated in developing countries, the major advantage of bananas being the expression of sufficient levels of antigenic protein in fruits (Kumar et al., 2005; Altindis et al., 2014).Nevertheless, leafy tissues entail major disadvantages, such as elevated water content, storage instability of harvested leaf biomass resulting in stability issues for the recombinant protein, and difficulties decoupling downstream and upstream processing. Therefore, the apparent advantage of leaf expression is offset by the likelihood of instability of the expressed proteins in tissues (and their extracts) that are metabolically active (Doran, 2006; De Muynck et al., 2010). Moreover, leaf extracts harbor phenolics, chlorophyll-derived pigments and inherent proteolytic activities that interfere with downstream processing (Yu et al., 2008; Woodard et al., 2009; Barros et al., 2011) resulting in laborious, multi-stage purification processes that impact the quantity and quality of the purified proteins. Further, despite the low costs associated with leafy and seed crops for production in open fields, biosynthesis of plant-based pharmaceuticals has moved on towards expression in confined environments such as bioreactors and greenhouses (McDonald et al., 2005; Spok and Karner, 2008).

### Downstream processing of plant-made recombinant proteins

Plant-made vaccines utilize plants as bioreactors for the production of vaccine antigens. This innovative approach offers several advantages over traditional vaccine production methods, such as lower production costs, scalability, and reduced dependence on cold chain storage (Habibi et al., 2017; Habibi et al., 2022). Protein purification, as an essential downstream technology in the field of biological industry, relies on defining an effective purification method and establishing continuous optimal strategy for refinement of purification steps. The objective is to achieve the desired concentration, purity, and yield of the target protein using the fewest possible steps. This process holds immense importance for subsequent research endeavors, providing researchers with high-quality proteins that can be suitable for diverse applications (Owczarek et al., 2019; Schiermeyer, 2020; Du et al., 2022).

**Figure 1:**
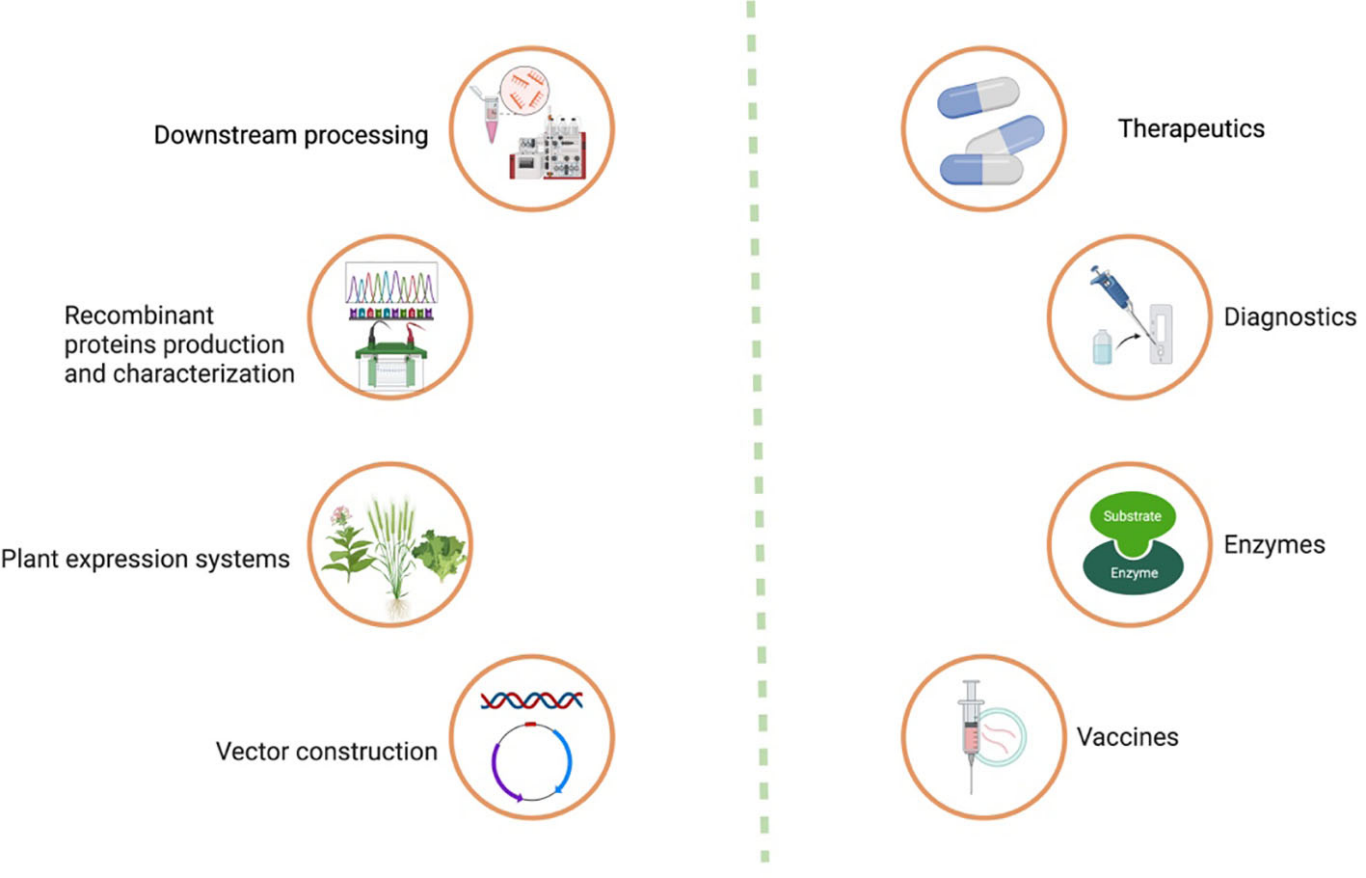
Process of recombinant protein production in plant system, its downstream processing, and applications.

**Figure 2:**
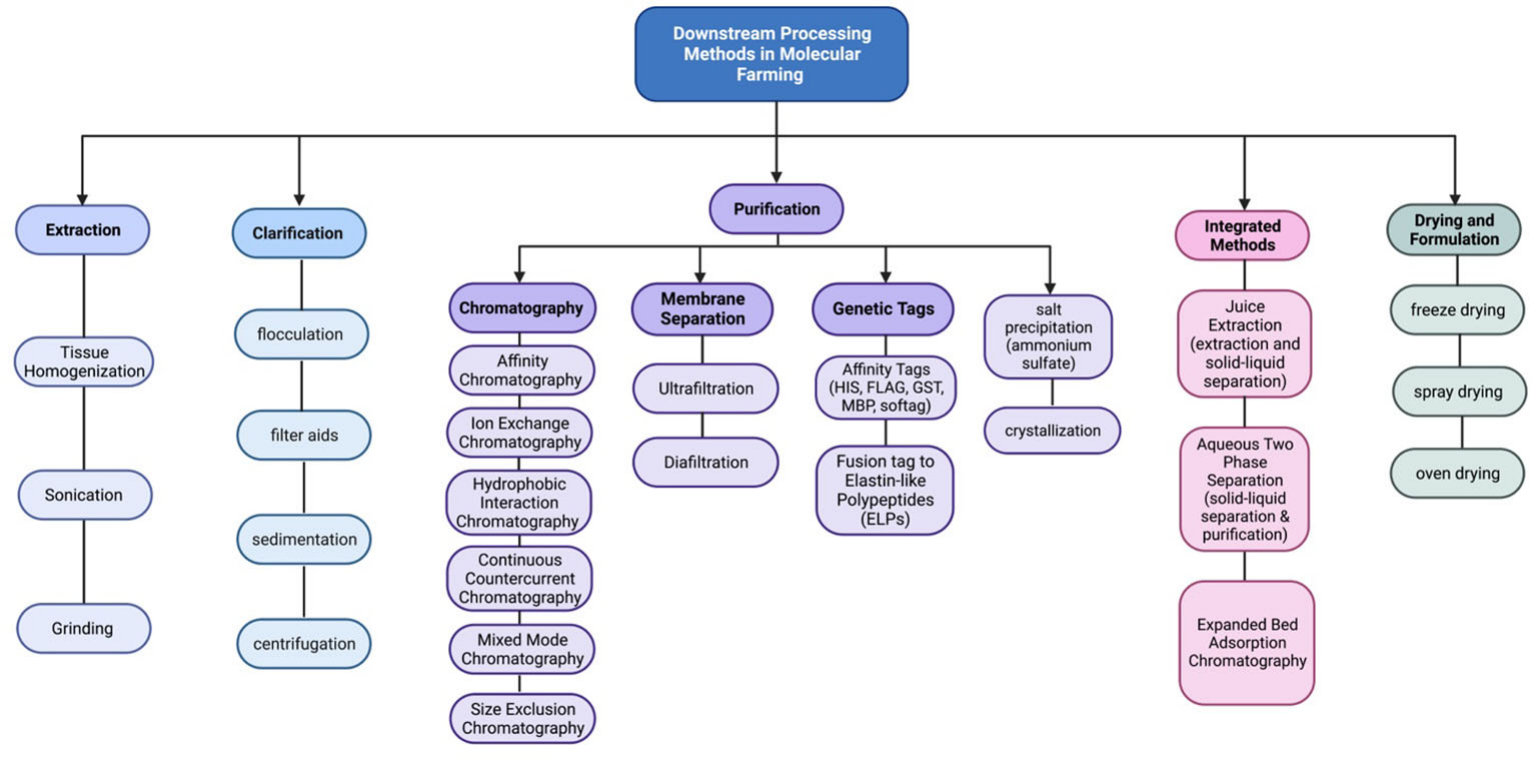
Schematic representation of the downstream processes used to clarify, concentrate, and purify recombinant proteins expressed in plant systems.

Moreover, purification strategies for plant-made vaccines are essential for ensuring the safety, efficacy, and regulatory compliance of innovative vaccines. The downstream processing process involves a series of steps which are shown in (**Figures 1**, **2**). Each step should be carefully designed to remove impurities, achieve high product purity, and maintain the integrity of the target antigen.

Prior to purification, it is beneficial to gather fundamental information about the target protein through bioinformatics analysis software. These analyses provide insights into various properties, including: isoelectric point (pI), cysteine content, protein stability, protein solubility, molecular weight (MW), secondary structure, susceptibility and sensitivity to oxidation and high amounts of salt ions or pH. Selecting an appropriate tag for facilitating overexpression and purification during vector construction is also important. Considerations such as the method for the purpose of cell disruption following protein expression and its potential impact on protein denaturation and structural changes should be addressed. These factors should be evaluated comprehensively to ensure optimal purification outcomes.

The purification approach for plant-derived proteins primarily involves leveraging the unique physical and chemical characteristics of the desired proteins. These properties include the sequence of a specific amino acid and its composition, the charge distribution, the polarity, and the hydrophilic or hydrophobic nature of the polypeptide chains, as well as the overall shape and the arrangement of amino acid residues present on the protein surface (Du et al., 2022). By carefully considering and understanding these characteristics, an effective purification strategy can be devised to isolate and purify the desired proteins in plant system.

The selection of a suitable purification method is crucial and should be based on not only careful consideration of the characteristics of the target protein but also the impurities. Here are some factors to take into account when making a reasonable selection:

(1) When considering the purification system for plant-based proteins, it is important to understand the impurity profile specific to plants. Some common impurities that can be encountered during plant protein purification are plant cell debris, soluble proteins, pigments and phenolic compounds, lipids and waxes, saccharides and polysaccharides and nucleic acids. These compounds can contribute to sample discoloration, sample turbidity, and affect protein stability and purity. Understanding the impurity profile specific to plant-based systems allows for the selection of appropriate purification strategies and optimization of purification conditions to effectively separate the target protein from unwanted components. Each purification step can be tailored to address these impurities, resulting in the isolation of high-quality plant proteins.
(2) Clarification, the initial step in purifying plant-based vaccines, serves as a crucial link between upstream and downstream processes. It has a crucial impact on determining the yield, consistency, and reproducibility of the final product. A well-executed clarification process should result in a solution with low turbidity while minimizing the impact on product recovery. It is designed to eliminate both process and product-related impurities. The clarification process should target the removal of larger particles, and insoluble and soluble impurities associated with the process or product, including large aggregates (Liu et al., 2010; Gupta et al., 2020). There are several clarification techniques commonly used in the purification of plant-based vaccines.
(3) Sedimentation: Sedimentation is a gravity-based clarification method where the plant extract can be allowed to stand undisturbed, allowing the larger particles and impurities to settle at the bottom due to their higher density or involves spinning the extract at high speeds to separate the heavier particles, such as plant cells, debris, and larger impurities, from the liquid phase. The clarified supernatant can then be collected, leaving behind the sedimented solids (Naik et al., 2012; Nejatishahidein et al., 2020).
(4) Filtration: Filtration methods, including depth filtration and microfiltration, are employed for both primary and secondary clarification of plant extracts for production of human papillomavirus (HPV) (Naupu et al., 2020) and hepatitis B virus (HBV) (Pantazica et al., 2023). The purification of these plant-derived vaccines often involves filtration steps to remove particulate matter, impurities, and host cell proteins while retaining the desired vaccine antigens. Depth filters, composed of fibrous or granular media, can effectively trap larger particles and impurities. Microfiltration membranes with pore sizes typically ranging from 0.1 to 10 micrometers are used to remove smaller particles and colloidal matter, resulting in a clarified solution.
(5) Flocculation can be employed to enhance the clarification of plant extracts (Buyel and Fischer, 2015). Flocculation is a process that involves the aggregation of fine particles and colloidal matter in a suspension, allowing them to form larger clumps or flocs. These flocs can then settle more readily or be easily separated through filtration, leading to improved clarity of the extract (Gregory and Barany, 2011; Buyel and Fischer, 2014b). Flocculants in plant extract clarification can include both natural and synthetic polymers such as Polyethyleneimine (PEI) (Zhang et al., 2013) polyacrylic acid (PAA;Wang et al., 2020) or polydiallyldimethylammonium chloride (PDADMAC)(Lin et al., 2007), Chitosan (Khodaei et al., 2018), and Aluminum Sulfate (Kittur et al., 2015; Park et al., 2015). In a study conducted by (Park et al., 2015). (Zhang et al., 2013), developed and evaluated a method by combining PEI precipitation and protein sample fractionation to improve Rubisco removal from TSP. By applying 50 and 100 mg/g of polyethyleneimine (PEI), a high efficiency of rubisco removal was achieved. Also, Ammonium sulfate was utilized for the purification of a plant-based colorectal cancer vaccine candidate GA733-FcK, and the researchers employed a concentration of 50% ammonium sulfate during the purification process. The results showed a significant 1.8-fold increase in the protein’s concentration. These polymers possess properties that promote the formation of flocs by bridging, charge neutralization, or adsorption mechanisms. By selecting an appropriate flocculant and optimizing the flocculation conditions (e.g., pH, temperature, flocculant dosage), the efficiency of the clarification process can be significantly improved (Buyel and Fischer, 2014a).
(6) Combination of Techniques: Often, a combination of clarification techniques is employed to achieve optimal results. For example, a sequence of centrifugation followed by filtration can be utilized to remove different sizes of particles and impurities (O’Leary et al., 2014; Sentis-Mor et al., 2022). The specific choice of clarification technique depends on factors such as the nature of the plant material, the desired level of purity, the scale of the process, and the properties of the target vaccine antigen. These clarification methods aid in the removal of unwanted particles, debris, and impurities from plant extracts, resulting in a clarified solution that can be further processed for downstream purification and formulation of plant-based vaccines.

Various chromatography techniques, such as AC, IEX, HIC, and SECb2 are commonly employed for purifying recombinant biopharmaceuticals. The main goal of these techniques is to attain a product of high purity, while ensuring its biological activity is preserved (Rathore et al., 2018). It is widely recognized that an elevated concentration of the target protein in the initial stages of the process requires a larger quantity of chromatography resin and an increased buffer demand.

AC is a widely recognized and highly selective technique used for purifying various plant-based biomolecules, including tagged proteins, bispecific antibodies, and vaccines (Fujita-Yamaguchi, 2015; Habibi et al., 2018; Song et al., 2019; Cibelli et al., 2022). Hexahistidine (His), glutathione S-transferase (GST), and maltose-binding protein (MBP) are among the commonly employed affinity tags. A notable challenge associated with Protein A chromatography is the leaching of Protein A, leading to binding of DNA, host cell proteins, and other impurities originated from the cell culture. These challenges involve increased resin expenses, restricted resin lifespan, modifications to the Protein A ligand, which necessitate exploration of alternatives including microspheres, and monolith membranes (Ramos-de-la-Pena et al., 2019). These endeavors aim to overcome the limitations associated with conventional Protein A chromatography, aiming for improved efficiency and cost-effectiveness in purification processes.

IEX is a highly utilized and cost-effective technique for purifying plant-based recombinant proteins (Champagne et al., 2013; Buyel and Fischer, 2014c). It involves cation CEX and AEX, which effectively remove various impurities including leached Protein A, media components, product variants, host cell proteins (HCPs) and DNA. HIC capitalizes on the diverse hydrophobic nature of protein molecules and is used as a critical part in the refinement of proteins. By HIC, proteins adhere to chromatographic ligands under high ionic strength conditions, while their release occurs under low ionic strength conditions. This technique efficiently segregates and purifies proteins based on their hydrophobic properties, thereby enhancing the overall purification process.

In SEC, protein molecules are separated based on their molecular weight. This approach has been widely employed for the purification of different proteins, including SARS-CoV-2 spike trimer vaccine (Song et al., 2019), human interleukin-6 (hIL6) (Islam et al., 2019), recombinant allergens and hypoallergenic variants (Weber et al., 2003).

In addition to SEC, membrane-based chromatography technique is used to achieve recombinant proteins with high purity. This approach involves attaching a high specific ligand to microfiltration membrane. In this method impurities can be removed from solution with neutral to basic pH as well as low conductivity. In this method however, optimizing flow distribution as well as the size and thickness of membrane are required for the purpose of protein purification (Orr et al., 2013; Chen et al., 2023). These advancements in membrane-based chromatography provide enhanced purification capabilities and broaden the options available for efficient protein purification processes.

### Examples of plant-made vaccine purification using affinity tags

Affinity tags are critical for bench-top purification as well as for characterization of the recombinant protein. However, not every purification tag may be economically viable at the production scale as removal of the tags (necessary for biopharmaceuticals) by chemical or enzymatic cleavage decreases the yield of the target protein and thus are not efficient.

Protein fusions are advantageous during plastid-based expression as the fusion partners stabilize and shield the chloroplast-expressed recombinant proteins from proteolytic degradation and enable fusion protein purification (Daniell et al., 2005). Nevertheless, fusion proteins expression level of > 10% of the total soluble protein form inclusion bodies that need solubilization followed by refolding before purification. Therefore, despite great advances in plastid transformation and recombinant protein expression, few reports exist on purification development using plastid-based platforms.

The fusion partner in fusion proteins can be advantageous for protein accumulation and facile purification. The GUS-interferon (INF) fusion protein was purified by weak anion-exchange chromatography and subsequent IMAC-Ni chromatography providing a yield of 6% of the TSP. The inclusion of His-tagged GUS as fusion enhanced expression 60-fold while simplifying purification. INF as fusion protein was cleaved off using the factor Xa with 58% efficiency followed by purification through cation-exchange chromatography (Leelavathi and Reddy, 2003). Two precipitation steps using ammonium sulfate and an IgG-affinity column were used to purify insulin-like growth factor (IGF) in fusion with the Z-domain of Staphylococcus aureus (Daniell et al., 2009). In this case, the fusion protein (~10% TSP) was subjected to chemical cleavage with hydroxylamine to remove the Z-domain. The cholera toxin B-proinsulin fusion that accumulated in the leaves of tobacco plants as inclusion bodies (up to 47% of TSP), was subjected to solubilization and refolding prior to purification by an IMAC-Ni column metal affinity chromatography as the cholera toxin B domain possesses three adjacent His residues that allow strong interactions between the Ni-agarose resin and the fusion protein (Boyhan and Daniell, 2011).

Santoni et al., 2022 propound the use of transient expression of recombinant proteins containing 6 His-tag fused to the N- or C-terminus of the recombinant vaccine candidate wherein the tag enables facile initial evaluation of the plant-derived candidate vaccine. However, the His tag must be removed in the final vaccine product prior to promoting it to the stage of clinical trials. Also, the appropriate positioning of the His tag has to be assessed considering that the position of the tag can influence protein accumulation and expression (Pinnola et al., 2015).

In both stable and transient expression systems, elastin-like polypeptide (ELP) derivatives (ELPylation) have been proven to enhance protein expression (Patel et al., 2007; Floss et al., 2010; Phan et al., 2014). The ELP tag can be used in the purification of fusion proteins (Phan and Conrad, 2016) which is particularly advantageous for veterinary applications with the necessity for low priced production. This is highly favorable for the production of vaccines needed for ensuring animal health, and it is a critical goal for producers driven by animal care regulations and the necessity to preclude contaminated food due to public health concern. Outbreaks of zoonotic disease such as swine flu and avian flu in the last few years have underlined the necessity of developing efficacious, scalable vaccination procedures (Organization, W. H., 2022). Trimerization of hemagglutinin (HA), the major flu antigen, is an essential tool to accomplish sufficient antigenicity (Cornelissen et al., 2010). ELPylation has been shown to augment expression while enabling scalable and low-cost purification also for HA trimers (Phan and Conrad, 2011; Phan et al., 2013b).

(Kim et al., 2021) cloned a fusion gene of the tumor-associated antigen GA733 glycoprotein (that is distinctly expressed in colorectal cancer) and the immunoglobulin Fc fragment (GA733-Fc), as well as the fusion of GA733-Fc with an endoplasmic reticulum retention motif (GA733-FcK) into the transient, deconstructed plant expression vector, pEAQ-HT derived from Cowpea mosaic virus. Both the fusion clones were transformed into *Agrobacterium tumefaciens*, followed by infiltration of the transformed Agrobacteria into the leaves of *Nicotiana benthamiana* plants. Following the identification of their maximal expression levels in the top leaves, both the fusion proteins were purified from the infiltrated leaves using protein A affinity chromatography. Shen et al., 2023 selected a SARS-CoV-2 spike protein-targeted human heavy chain variable domain (VHH) antibody fragment for rapid expression using plant cell suspensions and transgenic tobacco plants. Purification of this antibody was enabled through 6x His tag and C-Myc tag. This plant-derived VHH antibody proved to be capable of recognizing the SARS-CoV-2 spike protein as efficiently as that expressed in mammalian and bacterial cell cultures.

### Glycoengineering in plants

The majority of biologically significant proteins with therapeutic applications undergo N-linked glycosylation, and the sugar molecules within them significantly influence their folding, assembly, solubility, and functionality (Loos and Steinkellner, 2014). Consequently, glycoengineering, a process that alters the carbohydrate components of proteins to achieve specific protein characteristics, serves as an approach to enhance the effectiveness, safety, and resilience of pharmaceutical proteins. The difficulty lies in creating biological structures that can reliably generate homogenous glycan-containing glycoproteins on demand (Sethuraman and Stadheim, 2006). The accessibility of such structures will spur advancements on two levels: (i) understanding the role of sugar moieties in a variety of biological processes; and (ii) designing innovative biological substances with precisely customized glycosylation to fulfill their functional requirements (Chen, 2016).

The majority of eukaryotic cells have similar N-glycans pattern until the formation of the intermediate GnGn. However, processing after this point varies greatly, resulting in the synthesis of several complicated N-glycoforms. Glycoengineering is the most intriguing feature of plant-based systems for biopharmaceutical development. **Table 1** enlists some benefits and drawbacks of various expression host systems. Only two main glycan structures, GnGnXF and MMXF, are produced by plant cells, compared to mammals’ much reduced array of Golgi-located glycoenzymes (Varki, 2011) As a consequence, plant proteins often possess an unique dominating N-glycan structure as opposed to the variety of N-glycans found in CHO cell-derived proteins. Core 1,3-fucose and xylose, which are absent from human glycoproteins, are found in GnGnXF and MMXF (Loos and Steinkellner, 2014). There have been worries that plant-based proteins may set off immunological reactions that might end up in the induction of plant-glycan-specific antibodies, which might have negative repercussions. In contrast to mammalian cells, which have a massive glycome and leading to glycan variability that makes it difficult to specifically manipulate the N-glycosylation pathway, plants, offer a small repertoire of glycoenzymes for N-glycosylation, which enable plant system as ideal system to produce proteins with homogeneous glycans Chen, 2016}. Additionally, plants have a tremendous resistance for different glycan modifications, which cannot impact their growth or development phenotypes significantly.

Following this development, various well-defined human N-glycan structures, such as those featuring 1,6-fucosylation, bisected patterns, tetra-antennary structures, and bigalactosylation, have been effectively manufactured within plants (Loos and Steinkellner, 2014). As a result, CHO cells are no longer incapable of generating multi-branched N-glycans (Boune et al., 2020), all thanks to the innovation of glycoengineered plants. These plants can produce monoclonal antibodies (mAbs) with identical N-glycan profiles, differing only in their core 1,6-fucose (Yamane-Ohnuki and Satoh, 2009). These studies have also demonstrated that achieving the targeted production of human glycoforms necessitates the precise localization of the introduced glycoenzymes within specific subcellular compartments. Introducing mammalian enzymes randomly would disrupt the natural glycosylation pathway, leading to suboptimal or unusual hybrid N-glycans (Barolo et al., 2020). By employing a temporary expression method, this knowledge has facilitated the successful production of biantennary sialylated N-glycans by simultaneously expressing and accurately directing six different mammalian glycoenzymes to various subcellular locations (Loos and Steinkellner, 2014).

Another biosynthetic capability of plant systems is the production of terminal polysialic acid (polySia) glycoproteins, which can address challenges associated with the existing polySia conjugation process, such as the need for numerous fermentations, product purification, and *in vitro* chemical interactions (Colley et al., 2014).

PolySia plays multiple roles in various biological processes, such as cell regeneration, various immunological processes, and brain development Chen, 2016. By cotransforming six human glycoenzymes into XF plants, (Kallolimath et al., 2016) have successfully generated t plants that are capable of producing specific sialylated N-glycan structures with functional activity. The COVID-19 vaccine and taliglucerase-alpha serve as notable examples of the immense potential of using plant systems to produce glycosylated biopharmaceuticals. Recent research on the efficient production of vaccine candidates in low- and middle-income countries highlights the future advancements in plant-based glycosylated biopharmaceuticals. This research, as indicated by (Margolin et al., 2023) and (Phoolcharoen et al., 2023) suggests that manufacturing these molecules in plant systems can offer solutions to current challenges such as unequal vaccine distribution, high costs per dose, and the need for cold chain infrastructure, as discussed by (Chung et al., 2022).

Although plant glycoengineering has advanced significantly over the past 15 years, there are still several targets that need to be designed to minimize N and O-glycan variability and prevent potentially immunogenic glycan epitopes. These features are recently reviewed elsewhere by (Strasser, 2023) **Table 1**.

**Table 1:** Advantages and disadvantages of different expression host systems.

Host System	Pros	Cons
**Bacterial**	• Low cost in terms of culture condition (media and additives) • Require a short time to express recombinant proteins • The methods adapted to scale-up bioproduction are straightforward	• Large proteins are often highly variable in terms of expression and proteolyzed upon purification • The accumulation of inclusion bodies and protein precipitation Incorrect folding for large proteins, aggregation, or low chaperone activity • Endotoxin accumulation • No posttranslational modification for human proteins
**Mammalian**	• Post translational modifications • Correct protein folding • Human-ike glycosylation • Secretion capability • Scalability • Exiting regulatory approval	• Higher media and facility costs Longer cultivation times • The need for specialized equipment and expertise • Lower growth rates, Regulatory requirements and quality control measures, • Higher risk of human pathogen • large-scale mammalian cell culture can be challenging and costly
**Plants**	• Require less expensive growth media and facilities • Large scale protein production • Performing many post-translational modifications including glycosylation and disulfide bond formation • Low risk of contamination with human pathogens • Production of recombinant proteins in a relatively short time frame • No risk of mammalian pathogens Environmental friendliness.	• Protein yield variability • Transgene containment • Non-human glycosylation • Lacks regulatory approval
**Yeast**	• Rapid timeframe for the expression of recombinant proteins • Vectors are not dependent on helper viruses express • Secretion of most complex posttranslational modification proteins • Low secretion of host proteins • Existing regulatory pathway	• Glycosylation differences • Time-consuming • Scale-up challenges • More expensive than microbial systems due to the need for specialized media and insect cell lines

## Drawbacks of recombinant protein expression and purification in plant systems

### Effects of proteases on recombinant proteins expressed in plants

Plant cells have an abundance of proteolytic enzymes belonging to diverse classes (Rawlings et al., 2014; 2016) and are highly expressed in the lytic vacuole as well as the apoplast (Goulet et al., 2012). N-glycosylation (Saint-Jore-Dupas et al., 2007), and proteolytic processing directly influence the posttranslational fate of many heterologous proteins expressed in plants in addition to impacting the quality of the respective recombinant proteins (Pillay et al., 2014; Doran, 2006; Benchabane et al., 2008; Duwadi et al., 2015). Proteases play a primary role in controlling the turnover of proteins and are involved in the regulation of several developmental and cellular processes (Moon et al., 2004; Schaller, 2004; Smalle and Vierstra, 2004). Nevertheless, these enzymes are ubiquitous in plant tissues while being highly diverse (Grosse-Holz et al., 2018b; Beers et al., 2004) and pose a significant hurdle that compromises the successful expression of several recombinant proteins (Goulet and Michaud, 2006; Benchabane et al., 2008). Proteases are associated with several different aspects of the biological processes in plants including plant development, remobilization of nutrients, pathogen defense and senescence (Schaller, 2004; Liu et al., 2008; van der Hoorn, 2008). Proteases could impact the integrity of the expressed recombinant proteins in various ways, either within the plant following biosynthesis or outside the plant cell during the extraction procedures (Rivard et al., 2006; Benchabane et al., 2009a). Based on the number of sites susceptible and accessible to endogenous proteases for hydrolysis of peptide bonds, recombinant proteins could remain stable within the plant cell, or can be subjected to complete hydrolysis or partial cleavage that could negatively impact their activity, structural integrity or homogeneity and thereby their therapeutic value. Apart from negatively impacting the target protein yields, proteolytic processing could lead to generation of degradation products having equivalent physico-chemical characteristics as the intact recombinant target protein and hence difficult to get rid of during downstream extraction.

A plethora of recombinant proteins targeted to the apoplast have been expressed successfully in plants, but nevertheless, the plenitude and poor specificity of proteases within the apoplast often pose a major impediment that is not compatible with efficacious schemes of recombinant protein expression (Hellwig et al., 2004; Schiermeyer et al., 2005; Doran, 2006; Benchabane et al., 2008; Delannoy et al., 2008; De Muynck et al., 2009). Protease inhibitors have been used to augment recombinant protein expression in plants (Grosse-Holz et al., 2016; 2018a).

### Other approaches to improving recombinant protein accumulation

Other important parameters regarding the improvement of recombinant protein expression include maximizing or harmonizing codon preference as well as modifying tRNA pools towards the achievement of codon harmonization which requires prior knowledge of codon usage and the size of tRNA pools in the source organism and the host. Additionally, viral silencing suppressors could be co-expressed along with the recombinant protein to block systemic and local RNA silencing by precluding the accretion of siRNAs, disrupting siRNA-AGO interaction or eliciting AGO1 degradation (Watson et al., 2005; Baumberger et al., 2007). **Table 2** enlists some of the recombinant vaccines generated using various plant systems. Approaches to establishing ER stress resilience and alteration of degradation pathways could be added avenues for augmenting recombinant protein expression. Also, enhancing the protein storage capacity of the endomembrane system by expanding the ER through promotion of membrane synthesis serves to increase the productivity and capacity of the ER, thus overcoming ER stress (Schuck et al., 2009; de Ruijter et al., 2016; Vitale and Pedrazzini, 2005). Cells can be genetically engineered to synthesize larger quantities of phospholipids, especially phosphatidylcholine (PC) to facilitate increase in ER capacity.

**Table 2:** Recombinant vaccines produced in different plant systems.

Name	Function	Host	Expression	Reference
Hepatitis B Core Antigen (HBc)	Assembles into capsid particles, inducing immune response	*N. benthamiana*	2.38 g/kg FLW	(Huang et al., 2006)
Hepatitis B Surface Antigen (HBsAg)	Triggers induction of anti-HBs antibodies in humans following 2-3 doses	*Lactuca sativa L*. (transgenic lettuce), transformed with *A. tumefaciens*	>15 ng/g FLW (.0015mg/kg)	(Kapusta et al., 2001)
Respiratory Syncytial Virus F Protein (RSV-F)	Induction of Th-1 type response	Tomato *Lycopersicon esculentum*	1.0 - 32.5 µg/g FFW	(Sandhu et al., 2000)
Binding subunit of heat-labile enterotoxin in E coli (LT-B)	Triggers immune response and adds mucosal protection to intestine	Transgenic corn	Up to 10 mg/g (10 g/kg) in corn germ	(Tacket et al., 2004)
Rotavirus Capsid Protein (VP6)	Generates anti-VP6 Serum IgG and intestinal IgA antibodies; stimulates humoral and mucosal antibody production	Potato *Solanum tuberosum*	0.01% of TSP	(Yu and Langridge, 2003)
σC protein of avian reovirus, with strong promoter	Th-driven immune response	*Arabidopsis thaliana*	4.9% total soluble protein	(Wu et al., 2009)
Zika Virus Envelope Protein (ZIKV E)	Elicits cellular immune response and potent zE-specific antibody response	*N. benthamiana*	160 μg/g FW	(Yang et al., 2018)
Surface hemagglutinin in HAC1 influenza	Elicits HAI antibody response following 2 dosages, improved by presence of Alhydrogel (rabbits and mice)	*N. benthamiana*	90 mg/kg plant biomass	(Shoji et al., 2011)
Classical Swine Fever Virus E2 Subunit (CSFV - E2)	E2-specific antibody response	*N. benthamiana*	150 mg/kg plant biomass	(Park et al., 2020)
Pfs25 fused to ALfalfa mosaic virus coat for malaria vaccine	Production of serum antibodies that block malaria activity through binding of Pfs25	*N. benthamiana*	50 mg/kg	(Jones et al., 2013)
VHH nanobodies targeting SARS-CoV-2 spike protein	Production of single domain camel derived antibodies that bind CoV-2 spike protein	*N. tabacum* (suspension culture)	16.6 mg/mL solution (suspension culture)	(Shen et al., 2023)
Recombinant ESAT-6 + MPT-64 (tuberculosis antigens)	Recombinant protein fused by CTB subunit, used to promote polyclonal antibodies in rabbits	Transgenic cucumber	478 ng/g (0.03% v/v) TSP	((Yadav et al., 2023)
Human papillomavirus L1 protein under pentatricopeptide repeat target site	HPV-L1 triggers immune response; PPR protein drives expression by binding PPR sequence of mRNA to promote translation	*N. Tabacum*	0.03% TSP	(Legen et al., 2023)
Hepatitus E capsid protein containing M2e peptide of influenza A or receptor binding domain of SARS-CoV-2 spike protein	When expressed in plants, HEV Capsid protein assembles into virus-like particles that can be used as carriers of antigens (such as M2e)	*N Benthamiana*	M2e: 300-400 ug/g of fresh leaf tissue (10% TSP) SARS-CoV-2: 80-100 ug/g of fresh leaf tissue (1-2% TSP)	(Mardanova et al., 2022)
Chicken interleukin 17B	Immunoadjuvant for vaccine against chicken infectious bronchitis virus; enhances humoral and mucosal immune responses	*L. minor* ZH04043 (Duckweed)	1.89 ug/g FW, 0.0365% TSP	(Tan et al., 2022)
Colorectal carcinoma associated antigen (GA733-2)	Fruit used as vaccine, containing antigen that triggers immune response as well as intrinsic vitamins that help activate the immune system	*S. lycopersicum* (Tomato)	270 ng/g FW	(Park et al., 2022)

Plant storage vacuoles are preferred intracellular destinations for recombinant proteins expressed in seeds (Arcalis et al., 2014; Takaiwa et al., 2017; Torrent et al., 2009). Contrastingly, vacuoles in leaf cells as well as undifferentiated suspension cells are often deemed undesirable compartments for protein expression as they do not provide a stable environment. Despite this, several recombinant proteins such as endolysin, cellulolytic enzymes and avidin have been shown to accumulate at high levels in leaf central vacuoles (Marin Viegas et al., 2017). The human glucocerebrosidase was expressed in carrot cells using a vacuolar targeting signal that achieved the incorporation of the required N-linked glycan structure having terminal mannose residues, catalyzed by the activity of a glycan-modifying enzyme in the vacuole (Shaaltiel et al., 2007). Intriguingly, the plant-produced taliglucerase alfa, an acidic beta-glucocerebrosidase used in the treatment of Gaucher’s disease is capable of withstanding the highly hydrolytic conditions prevalent in the lysosome (Shaaltiel et al., 2007). Increase in recombinant protein expression could also be achieved through modulation of endogenous chaperone levels by overexpressing selected chaperones along with the recombinant protein of interest.

### Regulatory and safety issues concerning the expression and purification of vaccines/recombinant proteins in plants

During the production of plant-based vaccines, regulatory considerations are a vital component for approval of the recombinant product and therefore it is obligatory to follow specific guidelines as per good laboratory practices (GLPs) and good manufacturing practices (GMPs) enforced by regulatory agencies (Kirk et al., 2005; Tusé et al., 2020). Several heath organizations such as the International Council of harmonization (ICH) and the World Health Organization (WHO) covering regions in the United States, Japan and Europe play important roles in the development, implementation, surveillance and enforcement of regulatory strategies for the manufacture and administration of high-quality, effective and safe biopharmaceuticals in order to enable favorable public health outcomes (Tusé et al., 2020; European Medicine Agency (2008); WHO, 2005).

As per the FDA regulations of 2017, tobacco-derived recombinant products must get approval for use only upon fulfilment of the modified-risk tobacco products endorsement providing evidence concerning therapeutic information of the new plant-based product (Food and Drug Administration, HHS (2017)). Stringent rules and regulations regarding molecular farming are also enforced by the regulatory bodies of each specific country such as the ECCC in Canada and the USDAAPHIS in the USA primarily focused on precluding environmental risks concerned with the cultivation of plants producing recombinant vaccines (Hundleby et al., 2022). Tobacco has been shown to be a propitious system for the expression of plant-based vaccines because of its biocompatibility, low-cost, large-scale expression and low risk of dissemination of animal diseases. Nevertheless, protracted clinical trials have been hindered due to formidable regulatory and safety constraints (Mathew and Thomas, 2023).

The FDA has put forth stringent directives towards the safe application of plant-based products (FDA, 2020). Also, the European Medicines Agency (EMA) has decreed that herbal therapeutic compounds can only be administered if they have been used for a minimum of 30 years including at least 15 years within the EU as well as are not provided parenterally. Furthermore, use and marketing of these plant-derived products must be allowed only upon the availability of adequate scientific information confirming that the purified constituent or active ingredient of the plant-based product has recognizable potency (Calapai, 2008; EMA, 2020). Considering that there exist several toxic substances in plants, it is imperative that any side effects be ruled out due to unregulated administration of plant-derived products. Only then can plant-based vaccine candidates against diseases be considered for effectual use. Also, speedy harmonization of regulatory operations will enable reduction of the time span required for plant-based products to be adapted from the level of the bench to the market. Besides, plant-based vaccines must meet with best quality standards as per the strict GMP regulation guidelines assigned for all biological compounds. Despite the favorability of plant-based edible vaccines, there exist several challenges regarding their use. These include inappropriate gene transfer methodologies, low expression levels, impediments concerning codon bias and regulatory sites, improper polyadenylation, mRNA instability, epigenetic silencing, positional effects, inadequate immune response following administration, dose consistency variations and inappropriate selection of the vaccine antigen and host plant combination. The recent example of Medicago Inc. completed phase-2 clinical trials and also received regulatory approval of the candidate vaccine for SAR-Cov-2 by Health Canada will pave a way for plant-made products.

## Conclusions

As plant expression platforms evolve, a major focus of research and development will move from upstream to downstream processing, to increase overall productivity (Gottschalk, 2008). Downstream processing accounts for a major portion of the total costs for operation and product manufacturing. Hence, to be economically feasible, selective and efficient processes for product extraction and purification are increasingly called for (Basaran and Rodriguez-Cerezo, 2008). The efficiency of downstream processing is dependent on the concentration of the recombinant protein, complexities of cell-free culture media and plant extracts as well as the required level of purity of the final product. This review has sought to define the phases required to express and develop vaccines and other pharmaceutical proteins from plants. These steps will only become further refined as the search for cost-effective and environmentally friendly expression platforms for vaccines increase in demand.

## Author contributions

SV: Conceptualization, Supervision, Validation, Writing – original draft, Writing – review & editing, Investigation, Resources, Visualization. IK: Conceptualization, Formal analysis, Resources, Software, Supervision, Validation, Visualization, Writing – original draft, Writing – review & editing. PH: Conceptualization, Formal analysis, Project administration, Software, Supervision, Validation, Visualization, Writing – original draft, Writing – review & editing. ML: Validation, Writing – original draft, Data curation, Software. RL: Data curation, Software, Validation, Writing – original draft, Resources. KH: Validation, Writing – original draft, Conceptualization, Supervision, Writing – review & editing.
